# Assigned But Not Anchored: A Study of Primary Care Provider Utilization Among Covered California Enrollees

**DOI:** 10.1007/s11606-025-10111-w

**Published:** 2026-01-20

**Authors:** Barbara Rubino, Erik Taylor, Renée Vera Hagen

**Affiliations:** 1 Covered California, Health Equity and Quality Transformation Division, Sacramento, CA USA; 2 Covered California, Policy, Eligibility, and Research Division, Sacramento, CA USA

## Abstract

**Background:**

High-quality primary care is paramount for health equity and cost containment, yet many individuals lack access to a usual source of care. Since 2017, Covered California, the ACA Marketplace, has required all health plans to match enrollees with a PCP to serve as a first point of contact to promote high-value primary care.

**Objective:**

To understand primary care and overall healthcare utilization patterns for enrollees on ACA marketplace plans.

**Design:**

This study analyzed primary care utilization patterns across 1.1 million Covered California enrollees in 2022 under this policy and examined differences in usage of assigned and non-assigned PCPs across product types.

**Participants:**

In total, 1,112,292 Covered California enrollees aged 18 years or older in 2022.

**Main Measures:**

Our main measures included utilization rates for assigned primary care providers (PCPs), utilization rates of other PCPs, utilization of all non-primary care services, and rates of non-utilization of healthcare services.

**Key Results:**

Results indicated that while enrollees in PPO or EPO plans had higher overall rates of PCP utilization, differences in PCP usage disappeared after correcting for demographic and clinical differences between enrollees in HMO and PPO/EPO plans. However, visits to non-assigned PCPs are common in all plan types. And, 43% of adult enrollees with 12-months continuous enrollment did not have any primary care visits.

**Conclusions:**

We recommend that policy makers promote consistent utilization of any PCP regardless of PCP assignment, as we found that many people use other-than-assigned PCPs even when current policies intend to proactively steer them towards one main point of contact.

**Supplementary Information:**

The online version contains supplementary material available at 10.1007/s11606-025-10111-w.

## BACKGROUND

High-quality, accessible primary care is the key to driving equitable healthcare outcomes and containing costs.^1^ Prior research suggests that continuity of care with a primary care provider (PCP) who knows the patient is an important factor in lowering costs.^2–4^ Continuity of care is also associated with higher patient satisfaction.^5,6^ Yet, fewer people than ever have access to a usual source of care.^7,8^ When patients lose access to their usual PCP, healthcare costs increase and outcomes worsen.^4,9,10^

One way to support patients in accessing continuous primary care is to require that all people who enroll in health insurance select a primary care provider as the central point of contact for their healthcare needs. In 2017, Covered California, the Affordable Care Act (ACA) marketplace in California, implemented a universal requirement that all health plans match each enrollee with a PCP to serve as a “first point of contact and advocate” as a key element of its strategic approach to supporting high value primary care.^11^ Covered California currently also holds health plan issuers financially accountable to this universal PCP assignment requirement. This study explored the assigned and non-assigned PCP utilization patterns of enrollees in Covered California plans.

Health Maintenance Organization (HMO) plans typically require enrollees to select a PCP and require the PCP to authorize referrals to specialists.^6^ Preferred Provider Organization (PPO) and Exclusive Provider Organization (EPO) plans that largely use fee-for-service payment methods have traditionally been less prescriptive about primary care, and do not necessarily require an enrollee to select a PCP, nor do they require PCP authorization for specialist referrals. Enrollees in all Covered California plans must select a PCP and inform the plan of their PCP, and if they do not, the plan assigns them a PCP based on their location and the plan’s network. Under this policy, however, PPO/EPO plans do not require PCP authorization for specialty referrals; this requirement remains present only in HMO plans. Many other State Health Benefits Exchanges, as well as the other California public purchasers, California Public Employees Retirement System (CalPERS) and the California Department of Healthcare Services (DHCS), also require that all enrollees in all plans are assigned to a PCP.

Despite widespread requirements to assign all enrollees to PCPs, research on assigned and non-assigned PCP utilization in commercial health insurance (especially in PPO and EPO plans) has been limited. This study builds on existing research that has focused on the effect of gatekeeping models on utilization (see^12,13^ for reviews), utilization under non-universal assignment policies,^14^ and utilization of assigned PCPs in Medicaid plans in a closed system.^15^ Covered California requires all health plans, regardless of product type, to assign all members to a PCP if they have not selected one within 60 days of effectuation. Prior utilization, proximity, and panel status may be considered in health plan assignment methodology. In this study, we leveraged Covered California’s all-plan claims database to investigate whether enrollees in commercial HMO, PPO, and EPO plans receive care from their assigned PCP, and whether their use of PCPs differs between plan types under current universal PCP-assignment policies. With these data, we were able to identify consumers’ assigned PCP with high reliability, improving on common primary care-related reporting that relies on taxonomy^16^ or studies that infer assigned PCP based on past utilization.^14,17^ We compared the utilization of enrollees in HMO and PPO/EPO plans to investigate the following outcomes:

1. Use of the assigned PCP for primary care services
2. Use of any PCP for primary care services
3. Total number of PC visits with the assigned or any PCP

## METHODOLOGY

We used Covered California’s all-plan claims and enrollment database to investigate the association between plan type and healthcare utilization, including the use of the assigned and other PCPs.

### Data

The analytic data table was constructed out of three sets of information from Covered California’s administrative data: key enrollee and enrollment attributes for all 2022 enrollees (*n* = 2,317,869) (e.g. age, race/ethnicity, income level, plan type, issuer), PC visits (claims for assigned and other PCPs), overall utilization, total months enrolled, and a chronic condition flag denoting if the individual has any claims with a chronic condition diagnosis. Utilization data only included all paid claims that are approved by plan issuers, meaning that denied claims are excluded from our analysis.

PC services were identified based on procedure and provider taxonomy on the medical claims. We then used data from health plan issuers to determine whether a claim was made with the assigned PCP or another PCP (as identified exclusively by taxonomy).

We limited our analysis to enrollees aged 18 or older who were enrolled for 12 months, including those with no utilization during the year. In total, 11,041 (0.5%) of enrollees could not be matched with administrative data and are therefore excluded from our analyses. The resulting data set included 1,112,292 enrollees. In the supplemental materials, we show a replication of our analyses using the full population.

### Descriptive Analytics

Enrollees were assigned to one of four mutually exclusive and collectively exhaustive groups:

1. Utilized assigned PCP for primary care services (with any other utilization)
2. Utilized PCP other than assigned for primary care services (with any other utilization)
3. No use of any PCP for PC, but had other medical or pharmacy utilization.
4. No medical or prescription drug utilization of any type

To understand which of these four groups each enrollee fell into, we applied a hierarchical approach of assigning enrollees to the most specific group they fell into, even if they had visits with providers across groups. For example, if an enrollee saw only their assigned PCP, they were included in group 1. If an enrollee saw both their assigned PCP and another PCP, they were still included in group 1 as they had utilized their assigned PCP. If an enrollee saw their non-assigned PCP and had other medical or pharmacy utilization, they were counted as group 2. If an enrollee was seen only in an urgent care or emergency room, and had no utilization with any PCP, they would fall under group 3 (other medical or pharmacy utilization).

For the purposes of our analysis, we combined enrollees in PPO and EPO plans into one group (PPO/EPO) for two reasons. First, enrollees in both PPO and EPO plans do not require approval from their PCP to seek care with a specialist and are not required to seek care from a PCP as part of their health plan design. Second, enrollees in EPO plans account for less than 7% of total enrollees.

### Statistical Approach

To evaluate whether enrollees in HMO plans and enrollees in PPO or EPO plans differ in their usage of assigned PCPs and in overall PCP usage, we conducted several statistical tests. Using logistic regression, we first tested whether the enrollee’s plan type (HMO or PPO/EPO) affects the likelihood of having any PCP claims, and whether plan type affects the likelihood of having any PCP claims with the assigned PCP. In both models, plan type (HMO or PPO/EPO) is the sole predictor and claims are coded on a binary scale (0 for no claims, 1 for one or more claims). We repeated the above analyses using Zero-inflated Poisson models with the number of PCP claims with the Assigned PCP as the outcome and report the results in the Supplemental materials.

Second, we evaluated whether differences in claims between plan types may be partially explained by demographic differences between enrollees. Again, using logistic regression, we tested whether gender, race/ethnicity, household income (measured as percentage of the Federal Poverty Level (FPL), which is adjusted for family size), and an indicator for whether the individual has a chronic health condition were predictive of plan type (HMO or PPO/EPO). Third, we considered what effect of plan type on usage of any PCP remains after including these demographic and clinical characteristics as predictors to our model and report the average marginal effects (AME) of each predictor in this model. To estimate the variance explained by these demographic predictors, we compared the McFadden Adjusted Pseudo *R*^2^ of each of our models.

More detail on the statistical analyses can be found in the Supplementary materials. All statistical analyses were completed in R (version 4.1.3) with packages “tidyverse,” “stats,” “pscl,” and “marginaleffects,” and results were visualized using ‘ggplot2’.

## RESULTS

### All Population Descriptive Results

When we evaluated the 1.1 million adult enrollees with 12 months of continuous coverage in 2022, we observed that 58% of all enrollees had a primary care visit with any PCP: 30% had visits with their assigned PCP and 28% had visits with a PCP who was not their assigned PCP. In total, 43% of enrollees across all plan types had no primary care visits with any PCP. A total of 30% of enrollees had only non-primary care utilization, such as with a specialist or in an emergency room or filled a prescription drug, and the remaining 13% of enrollees had no healthcare utilization of any type. Enrollees in PPO/EPO plans who had PC visits were as likely to use another PCP as their assigned PCP. Comparing members enrolled in HMO and PPO/EPO plans, a higher percentage of the PPO/EPO population self-identified as White, and more members enrolled in PPO/EPO plans had a chronic condition, while more enrollees in HMO plans identified as Asian American or Hispanic or Latino. Incomes were on average lower among enrollees in HMO plans (Table 1).

**Table 1:** Demographic Characteristics of Adult HMO and PPO/EPO Enrollees Who Were Enrolled for 12 Months

	HMO	PPO/EPO
*N*	790,337	321,955
PCP and other health care utilization		
Used assigned PCP	288,627 (36.5%)	41,411 (12.9%)
Used other PCP	149,906 (19.0%)	161,361 (50.1%)
No PCP use, other heath care use	244,469 (30.9%)	85,519 (26.6%)
No utilization	107,335 (13.6%)	33,664 (10.5%)
Average number of PCP visits		
With assigned PCP	0.8 (1.5)	0.3 (1.2)
With other PCP	0.7 (1.4)	1.7 (2.6)
Age	45.5 (13.5)	45.9 (13.3)
Gender: Women	404,430 (51.2%)	169,665 (52.7%)
Chronic disease diagnosis	241,212 (30.5%)	141,488 (43.9%)
Race/ethnicity		
American Indian/Alaska Native	1,342 (0.2%)	1,321 (0.4%)
Asian American	187,920 (23.8%)	45,468 (14.1%)
Black or African American	17,154 (2.2%)	3,754 (1.2%)
Hispanic or Latino	195,592 (24.7%)	52,332 (16.3%)
Native-Hawaiian/Pacific Islander	803 (0.1%)	177 (0.1%)
White	187,788 (23.8%)	119,514 (37.1%)
Multi-racial	14,386 (1.8%)	6,231 (1.9%)
Other	45,588 (5.8%)	20,610 (6.4%)
Nonrespondent	139,764 (17.7%)	72,548 (22.5%)
Income (% of FPL)		
0 < = 138	12,185 (1.5%)	4,388 (01.4%)
138 < = 150	116,794 (14.8%)	40,302 (12.5%)
150 < = 200	233,110 (29.5%)	81,021 (25.2%)
200 < = 250	136,120 (17.2%)	47,346 (14.7%)
250 < = 400	192,448 (24.4%)	87,084 (27.0%)
400 +	65,175 (8.3%)	36,790 (11.4%)
Unsubsidized	34,505 (4.4%)	25,024 (7.8%)

The mean and standard deviation for age and number of PCP visits, and counts and percentages out of the total for other characteristics

Source: Authors’ analysis of Covered California’s administrative data

### Primary Care Provider Utilization by Plan Type

We investigated whether plan type (HMO, PPO/EPO) is associated with differences in use of assigned or other PCP. We found that enrollees in HMO plans were about 23.7 percentage points (95% CI 23.5–23.8) more likely to have at least one visit with their assigned PCP compared with enrollees in PPO/EPO plans (Table 2, Table S1).

**Table 2:** Model Coefficients of Two Logistic Regression Models, Predicting Whether Enrollees Had Any Claims with the Assigned PCP and with Any PCP by Plan Type

	Assigned PCP		Any PCP	
	Coefficient	CI 95%	Coefficient	CI 95%
Plan type: PPO/EPO	−1.360	[−1.372 to −1.349]	0.312	[0.303—0.320]
Constant	−0.553	[−0.558 to −0.548]	0.220	[0.215—0.224]
McFadden Adjusted Pseudo *R*^2^	0.050		0.004	

Source: Authors’ analysis of Covered California's administrative data

When we expanded our view to include utilization of any PCP, we found that enrollees in PPO/EPO plans were 7.5 percentage points (95% CI 7.3–7.7) more likely to have a visit with any PCP, either their assigned or another PCP (Table 2, Table S24). Enrollees in PPO plans had a higher total volume of PCP visits than did enrollees in HMO plans. In a year, enrollees in PPO/EPO on average had 0.53 (95% CI 0.53–0.54) more claims with any PCP compared with enrollees in HMO plans (Table S2, Table S4). We found similar results when expanding our analyses to also include children and individuals who were enrolled for less than 12 months (Table S5, Table S6).

### Utilization and Enrollee Characteristics

Subsequent analyses suggest that differences in demographic and clinical factors between enrollees in PPO/EPO plans versus HMO plans may account for variation in usage of PCPs between these groups. When we investigated differences among race/ethnicity groups in plan type, models adjusting for gender indicated that enrollees who identify as American Indian and Alaska Native (AIAN) (*β* = 0.637, 95% CI 0.560–0.713) and enrollees who identify as White (*β* = 0.205, 95% CI 0.193–0.217) were more likely to be enrolled in a PPO or EPO plan compared with individuals who identify as other race/ethnicity groups (e.g., for Black or African-American enrollees *β* = −0.864, 95% CI −0.901–−0.828, Table S7). We also found that the higher someone’s income, the more likely they were to be enrolled in a PPO/EPO plan (e.g., for incomes at 138–150% FPL, *β* = −0.044, 95% CI −0.081 to −0.008, compared with for 400% + FPL *β* = 0.465, 95% CI 0.428–0.503 after adjusting for gender and chronic disease diagnosis, Table S8). Furthermore, people with a chronic disease diagnosis were more likely to be enrolled in a PPO or EPO compared with those not diagnosed with a chronic disease (*β* = 0.595, 95% CI 0.588–0.605 after adjusting for income and gender, Table S8). 

A logistic regression model that included these factors as predictors to test whether enrollees have any claims with any PCP showed that the effect of plan type on PCP usage is strongly attenuated; the coefficient of plan type shrinks from 0.312 (95% CI 0.303—0.320, Table 2) in the base model to 0.061 (95% CI 0.052—0.071, Table 3) when enrollee characteristics were included. The McFadden Adjusted Pseudo *R*^2^ of this full model including enrollee characteristics was 0.132, compared to 0.004 for the base model. In the full model, chronic condition diagnosis had the largest average marginal effect (AME) on the predicted probability of having a PCP claim (0.404, 95% CI 0.403—0.406), compared with the relatively small AME of plan type (0.012, 95% CI 0.011—0.014) (Table S9). The presence of a diagnosed chronic condition was also the strongest contributor to the explained variance of this full model; after omitting chronic condition from the model, the McFadden Adjusted Pseudo *R*^2^ decreased to 0.013 (Table S10).

**Table 3:** Model Coefficients of a Logistic Regression Model Predicting Whether Enrollees Had Any Claims with Any PCP

	Coefficient	CI 95%
Plan type: PPO/EPO	0.061	[0.052—0.071]
Income group (percentage of FPL):		
138 < = 150	−0.075	[−0.11 to −0.039]
150 < = 200	−0.060	[−0.095 to −0.025]
200 < = 250	−0.079	[−0.115 to −0.044]
250 < = 400	0.019	[−0.016—0.054]
400 +	0.176	[0.139—0.213]
Unsubsidized	0.010	[−0.028—0.048]
Gender: Man	−0.448	[−0.456 to −0.439]
Race/ethnicity		
American Indian/Alaska Native	0.207	[0.119—0.294]
Asian	−0.118	[−0.131 to −0.105]
Black or African American	−0.007	[−0.039—0.025]
Hispanic or Latino	−0.026	[−0.039 to −0.013]
Multi-racial	−0.027	[−0.004—0.058]
Native Hawaiian/Pacific Islander	−0.189	[−0.328 to −0.05]
White	0.071	[0.059—0.084]
Other	0.084	[0.065—0.104]
Chronic diagnosis	1.951	[1.941—1.961]
Constant	−0.031	[−0.066—0.005]
McFadden Adjusted Pseudo *R*^2^	0.132	

This full model includes plan type, income as percentage of the FPL, age group, gender, race/ethnicity group and chronic disease diagnosis as predictors

Source: Authors’ analysis of Covered California administrative data

This full model also showed that income is associated with the likelihood of having PCP claims: the probability of having claims with any PCP was 5 percent points higher at incomes of more than 400% of the FPL compared with incomes of 138–150% of the FPL (0.0508, 95% CI 0.047—0.054). Men were about 9 percent points less likely to have any PCP claims compared to women (–0.092, 95% CI 0.094—0.090) (Table S9).

## DISCUSSION

While PCP assignment requirements are increasingly adopted outside of HMO plans, the relationship between PCP utilization and plan type in the commercial insurance market has not been thoroughly investigated. Our research finds that for enrollees in EPO/PPO plans and those in HMO plans, their assigned PCP often did not align with primary care utilization patterns. This was most pronounced amongst members enrolled in PPO/EPO plans, where only 13% of members used their assigned PCP and another 50% of members did not visit their assigned PCP and used another PCP. Although the design of HMO plans funnels care through an assigned PCP, we observed that members often did not utilize their assigned PCP. In total, 19% of enrollees in HMO plans did not have claims with their assigned PCP but did visit another PCP. These findings are similar to Love and colleagues,^15^ who observed that most people in a sample of Arizona’s Medicaid enrollees received treatment from someone other than their assigned PCP.

Regardless of whether an enrollee uses their assigned PCP or another PCP, they receive important preventive and disease management services. However, 43% of all adult enrollees with 12 months of continuous enrollment did not have any primary care visits.

Our analysis found that overall, enrollees in PPO/EPO plans are more likely to use PC services as compared to enrollees in HMO plans. Importantly, the differences in PCP usage seem to arise from demographic and clinical differences between populations, which may prompt individuals to select plans based on their planned or projected healthcare needs. This result is similar to the results of Barnett and colleagues,^16^ who find that among Massachusetts residents, HMO enrollees had fewer PCP visits, and that this difference largely disappeared after controlling for enrollee characteristics. Most notably, we found that more PPO/EPO enrollees are diagnosed with chronic medical conditions, and this seems to contribute heavily to the higher probability of claims among PPO/EPO enrollees.

Another notable finding of our study was the observed positive association between enrollee income and utilization, which is consistent with some earlier findings of cost sensitivity (although the evidence is mixed,^18,19^ see^20^ for contrasting findings). This association between income and utilization contributed to differences in claims between enrollees in HMO and PPO/EPO plans, with members in PPO/EPO plans having higher income levels. This finding is also remarkable on its own given that Covered California’s income-dependent subsidies considerably decrease out-of-pocket costs for low-income enrollees, which aim to mitigate the impact of cost on utilization. The links between income and utilization and income and plan choice could also result from consumers’ education or work status, factors that are unmeasured in our data but likely covary with income.

Finally, Covered California plans all have standard benefit designs across all plans within a metal tier. All preventive services are covered with no cost sharing to members regardless of metal tier.

### Study Limitations

A possible limitation of our study is that our indicator for chronic conditions is conditional on having approved claims in the calendar year. Those without PCP claims could still be marked in our data with a chronic condition diagnosis through a non-PCP medical claim, but we may have undercounted chronic condition prevalence among HMO enrollees, who have fewer claims on average. Our findings are consistent with other research observing healthier consumers and lower health care usage in HMOs,^14,21,22^ but in this observational study we were not able to properly assess the causal pathways between these factors. Future research could incorporate health risk scores for a more complete picture of health and disease burden in HMO and PPO/EPO populations. 

An additional study limitation is our inability to accurately identify the population of members who self-selected a PCP versus those who were assigned one. This potential stratification could enhance future analysis and better help us understand utilization.

## CONCLUSION

We recommend that policy makers promote consistent utilization of any PCP regardless of assignment, as we find that many people do not stay anchored to their assigned PCP, often using other PCPs even when current policies intend to proactively steer them towards one main point of contact. Because of these findings, Covered California is proposing new policies that promote greater use of primary care and continuity of care regardless of assignment as part of its 2026–2028 contract with health plans, including financial accountability associated with continuity of care reporting.^23^ We encourage other policy making and regulatory bodies across payer types and other states to expand policies beyond PCP assignment and encourage the utilization of primary care and incentivize continuity. Establishing a relationship with a known, trusted PCP is foundational to continuity of care, and whether that provider is the assigned PCP or not, the longitudinal relationship has been shown to reduce emergency room utilization,^2^ improve overall healthcare costs,^4^ and lead to equitable outcomes.^24^

## Supplementary Information

Below is the link to the electronic supplementary material.

Supplementary Material 1 (DOCX 125 KB)

## Data Availability

Data supporting this study cannot be made available due to proprietary nature of claims information submitted to Covered California's all-plan claims database. Further information on data supporting this study is available in the Supplementary Materials.
