# Supplementary material for: Assigned But Not Anchored: A Study of Primary Care Provider Utilization Among Covered California Enrollees
Source: J Gen Intern Med. 2026 Jan 20;41(11):3049–54. doi: 10.1007/s11606-025-10111-w (PMC13493671; doi:10.1007/s11606-025-10111-w)
Supplement: Supplementary file 1 [file 11606_2025_10111_MOESM1_ESM.docx]

**Supplementary Materials**

*Data*

Income as percentage of the Federal Poverty level (FPL): An individual's household income as percentage of the FPL is calculated when they apply for coverage through Covered California. The FPL with which their income is compared is dependent on household size. A small percentage of enrollees applies for unsubsidized coverage. These individuals do not have to supply their income and are therefore categorized as ‘Unsubsidized’.

*Methods*

We predicted the binary outcome of having any claims with the Assigned PCP or with any PCP with two logistic regression models:

$$Y_{i}\sim Binomial\left( 1, p_{i} \right)$$

$$\log it\left( p_{i} \right)= I +\beta_{1}\cdot Plantype_{i}$$

In addition to the logistic regression models reported in the main paper, we used Zero-inflated Poisson regression to model the number of claims per enrollee depending on their plan type. We again used separate models the predict number of claims with the Assigned PCP and the number of claims with any PCP. Both the binary and count parts of these models included only plan type as predictor:

$$Y_{i}\sim ZIPoisson\left( p_{i}, \lambda_{i} \right)$$

$$\log it\left( p_{i} \right)= I +\beta_{1}\cdot Plantype_{i}$$

$$\log it\left( \lambda_{i} \right)= I +\beta_{1}\cdot Plantype_{i}$$

To investigate how our choice to include only adults who were enrolled for the full 12 months affected our results, we also ran these models with the full data set. Here the logistic part of each model includes predictors for plan type (HMO or PPO/EPO), enrollment length (in months) and an interaction effect between plan type and enrollment length. The count parts of these models are adjusted for exposure by using logged enrollment length as offset:

$$Y_{i}\sim ZIPoisson\left( p_{i}, \lambda_{i} \right)$$

$$\log it\left( p_{i} \right)= I +\beta_{1}\cdot Plantype_{i}+\beta_{2}\cdot Months_{i}+\beta_{3}\cdot Plantype_{i}\cdot Months_{i}$$

$$\log\left( \lambda_{i} \right)=\log\left( \frac{\mu_{i}}{Months_{i}} \right)= I +\beta_{1}\cdot Plantype_{i}$$

The full model predicting the binary outcome of having any claims with any PCP uses this formula:

$$Y_{i}\sim Binomial\left( 1, p_{i} \right)$$

$$\log\left( p_{i} \right) =I +\beta_{1}\cdot Plantype_{i}+\beta_{2}\cdot FPL{138}_{i}+\beta_{3}\cdot FPL{150}_{i}+\beta_{4}\cdot FPL{200}_{i}+ \beta_{5}\cdot FPL{250}_{i}+ \beta_{6}\cdot FPL{400}_{i}+ \beta_{7}\cdot FPLunsub_{i}+ \beta_{8}\cdot Male_{i}+\beta_{9}\cdot AIAN_{i}+\beta_{10}\cdot Asian_{i} + \beta_{11}\cdot Black_{i}+\beta_{12}\cdot Latino_{i}+\beta_{13}\cdot NHPI_{i}+ \beta_{14}\cdot Latino_{i}+\beta_{15}\cdot White_{i}+\beta_{16}\cdot Other_{i}+\beta_{17}\cdot Chronic_{i}$$

McFadden's (1974) Adjusted Pseudo R2 is used to compare model fit. This R2 penalizes models for any predictors with little explanatory value by adjusting for K number of predictors:

$$R_{adj}^{2}= 1 - \frac{\ln L\left( M_{full} \right)-K}{\ln L\left( M_{intercept} \right)}$$

**Results**

*Effect of plan type on having the number of PCP claims*

Investigating whether the number of claims per enrollee varied depending on plan type (HMO or PPO/EPO), we conducted zero-inflated Poisson regression models. These results were in line with the results from our binary models: HMO enrollees have a higher number of claims with their assigned PCP (Table S1, Table S3), while PPO/EPO enrollees have more claims when considering any PCP (Table S2, Table S4).

| Count model coefficients (Poisson with log link): | |
| --- | --- |
| Plan type: PPO / EPO | 0.175 [.167 - .183] |
| Constant | 0.643 [.640 - .646] |
| Zero-inflation model coefficients (Binomial with logit link): | |
| Plan type: PPO / EPO | 1.502 [1.490 - 1.514] |
| Constant | 0.285 [.279 - .290] |

Table S1. Model coefficients of a Zero-Inflated Poisson regression predicting the number of claims with the Assigned PCP by plan type.

| Count model coefficients (Poisson with log link): | |
| --- | --- |
| Plan type: PPO / EPO | 0.218 [.214 - .222] |
| Constant | 0.910 [.907 - .912] |
| Zero-inflation model coefficients (Binomial with logit link): | |
| Plan type: PPO / EPO | -0.235 [-.245 - -.226] |
| Constant | -0.427 [-.432 - -.422] |

Table S2. Model coefficients of a Zero-Inflated Poisson regression predicting the number of claims with the any PCP by plan type.

*Predicted probability of claims and number of claims by plan type*

| Plan type | Predicted probability of having at least one claim [CI 95%] | Predicted number of claims [CI 95%] |
| --- | --- | --- |
| HMO | .365 [.364 - .366] | .816 [.813 - .819] |
| PPO / EPO | .129 [.127 - .130] | .325 [.322 - .328] |

Table S3. Predicted probability of at least one claim and number of claims with the assigned PCP for adult enrollees with 12 months continuous enrollment by plan type.

| Plan type | Predicted probability of having at least one claim [CI 95%] | Predicted number of claims [CI 95%] |
| --- | --- | --- |
| HMO | .555 [.554 - .556] | 1.50 [1.500 — 1.507] |
| PPO / EPO | .630 [.628 - .631] | 2.04 [2.032 — 2.046] |

Table S4. Predicted at least one claim and number of claims with any PCP for adult enrollees with 12 months continuous enrollment by plan type.

*Effect of plan type on having any PCP claims with the full dataset*

We checked how robust our findings are to our choice to limit the data to only adults who were enrolled the full year. When we include all enrollees regardless of age or enrollment length, and adjust the models by enrollment year (see methods section), the effect of PPO/EPO on assigned PCP claims is similar to the effect found for 12-months enrollees (Table S5). The effect of PPO/EPO on any PCP claims is smaller than in the model limited to 12-months enrollees, but remains positive (Table S6).

| Enrollment length (months) | 0.191 | [.190 - .192] |
| --- | --- | --- |
| Plan type: n | -1.148 | [-1.182 - -1.113] |
| Enrollment length (months) * PPO / EPO | -0.020 | [-.023 - -.017] |
| Constant | -2.788 | [-2.800 - -2.777] |
| McFadden Pseudo R^2^ | 0.110 | |

Table S5: Model coefficients of a logistic regression model predicting whether enrollees had any claims with the Assigned PCP by plan type. Including all enrollees regardless of enrollment length.

| Enrollment length (months) | 0.203 | [.203 - .204] |
| --- | --- | --- |
| Plan type: PPO / EPO | 0.108 | [.091 - .125] |
| Enrollment length (months) * PPO / EPO | 0.020 | [.018 - .021] |
| Constant | -2.135 | [-2.144 - -2.126] |
| McFadden Pseudo R^2^ | 0.118 | |

Table S6: Model coefficients of a logistic regression model predicting whether enrollees had any claims with any PCP by plan type. Including all enrollees regardless of enrollment length.

*Demographic differences in plan type*

We tested whether we observe an effect of race/ethnicity of plan type (with coding HMO = 0, PPO/EPO = 1), controlling for age and gender. We find large differences between race/ethnicity groupings in the likelihood the be enrolled in an HMO or PPO/EPO, with white and AIAN consumers more likely to be enrolled in a PPO/EPO plan, whereas Black or African American, Hispanic or Latino, Asian and Native Hawaiian/Pacific Islander consumers are more likely to be enrolled in an HMO (Table S7). We also examined the likelihood of being enrolled in an HMO or PPO/EPO for consumers who are diagnosed with a chronic illness, controlling for income, age and gender. This same model allows us to simultaneously test the likelihood of enrollment in an HMO or PPO/EPO depending on income, controlling for chronic illness, age and gender. The results indicate that individuals with a chronic illness diagnosis and wealthier consumers are more likely to be enrolled in a PPO/EPO plan (Table S8).

|  | Coefficient | CI 95% |
| --- | --- | --- |
| Race / ethnicity: |  |  |
| American Indian/Alaska Native | 0.637 | [.560 - .713] |
| Asian | -0.764 | [-.778 - -.750] |
| Black or African American | -0.864 | [-.901 - -.928] |
| Hispanic or Latino | -0.665 | [-.678 - -.651] |
| Multi-racial | -0.181 | [-.212 - -.150] |
| Native Hawaiian/Pacific Islander | -0.857 | [-1.020 - -.694] |
| White | 0.205 | [.193 - .217] |
| Other | -0.138 | [-.1556 - -.119] |
| Gender: man | -0.077 | [-.085 - -.069] |
| Constant | -0.618 | [-.628 - -.609] |

Table S7. Model coefficients of a logistic regression model predicting whether enrollees are enrolled in a PPO/EPO (as opposed by HMO) depending on their race/ethnicity group, controlling for age and gender.

|  | Coefficient | CI 95% |
| --- | --- | --- |
| Chronic diagnosis | 0.597 | [.588 - .605] |
| Income group (percentage of FPL): |  |  |
| 138 <= 150 | -0.044 | [-.081 - -.008] |
| 150 <= 200 | -0.031 | [-.067 - .005] |
| 200 <= 250 | -0.021 | [-.060 - 0.015] |
| 250 <= 400 | 0.248 | [.213 - .285] |
| 400+ | 0.465 | [.428 - .503] |
| Unsubsidized | 0.760 | [.721 - .799] |
| Gender: man | -0.052 | [-.060 - -.043] |
| Constant | -1.231 | [-1.267 - -1.196] |

Table S8. Model coefficients of a logistic regression model predicting whether enrollees are enrolled in a PPO/EPO (as opposed by HMO) depending on their chronic illness status and income, controlling for age and gender.

| Term | Contrast | Estimate | Statistic | CI 95% |
| --- | --- | --- | --- | --- |
| Chronic diagnosis | Chronic | 0.404 | 485.779 | [.403 - .406] |
| Income group | 138<=150 | -0.015 | -4.106 | [-.022 - -.008] |
| Income group | 150<=200 | -0.012 | -3.394 | [-.019 - -.005] |
| Income group | 200<=250 | -0.016 | -4.4 | [-.023 - -.009] |
| Income group | 250<=400 | 0.004 | 1.051 | [-.003 - .011] |
| Income group | 400+ | 0.036 | 9.381 | [.028 - .043] |
| Income group | Unsubsidized | 0.002 | 0.521 | [-.006 – .010] |
| Gender | Man | -0.092 | -106.941 | [-.094 - -.090] |
| Plan type | PPO / EPO | 0.012 | 12.633 | [.011 - .014] |
| Race/ethnicity | AIAN | 0.042 | 4.652 | [.024 - .059] |
| Race/ethnicity | Asian | -0.024 | -17.672 | [-.027 - -.021] |
| Race/ethnicity | Black or African American | -0.001 | -0.432 | [-.008 - .005] |
| Race/ethnicity | Hispanic or Latino | -0.005 | -3.87 | [-.008 - -.003] |
| Race/ethnicity | Multiple races | 0.006 | 1.705 | [-.001 - .012] |
| Race/ethnicity | NHPI | -0.039 | -2.658 | [-.067 - -.01] |
| Race/ethnicity | Other | 0.017 | 8.545 | [.013 - .021] |
| Race/ethnicity | White | 0.014 | 11.331 | [.012 - .017] |

Table S9. Average marginal effects of each predictor variable in the full model. Reference values are as follows: Chronic: No chronic diagnosis, income: 0<=138, plan type: HMO, gender: woman, race/ethnicity: non-respondent.

| Variable removed | McFadden Adjusted Pseudo R^2^ |
| --- | --- |
| None (Full model) | .1320 |
| Plan type: PPO / EPO | .1319 |
| Income | .1312 |
| Race/ethnicity | .1312 |
| Gender | .1244 |
| Chronic diagnosis | .0132 |

Table S10. McFadden Adjusted Pseudo R^2^ for the full model after removing one variable at a time.

*Definition of Chronic Conditions*

A member was identified as having a chronic condition in claims if they had a diagnosis code from the list below lasting 12 months or more and requiring ongoing medical attention.

| **Diagnosis Code** | **Description** |
| --- | --- |
| A419 | Sepsis, unspecified organism |
| B181 | Chronic viral hepatitis B without delta-agent |
| B182 | Chronic viral hepatitis C |
| B1910 | Unspecified viral hepatitis B without hepatic coma |
| B20 | Human immunodeficiency virus [HIV] disease |
| C187 | Malignant neoplasm of sigmoid colon |
| C189 | Malignant neoplasm of colon, unspecified |
| C20 | Malignant neoplasm of rectum |
| C3490 | Malignant neoplasm of unspecified part of unspecified bronchus or lung |
| C439 | Malignant melanoma of skin, unspecified |
| C44311 | Basal cell carcinoma of skin of nose |
| C44319 | Basal cell carcinoma of skin of other parts of face |
| C44329 | Squamous cell carcinoma of skin of other parts of face |
| C4441 | Basal cell carcinoma of skin of scalp and neck |
| C44519 | Basal cell carcinoma of skin of other part of trunk |
| C44529 | Squamous cell carcinoma of skin of other part of trunk |
| C44612 | Basal cell carcinoma of skin of right upper limb, including shoulder |
| C44619 | Basal cell carcinoma of skin of left upper limb, including shoulder |
| C44622 | Squamous cell carcinoma of skin of right upper limb, including shoulder |
| C44629 | Squamous cell carcinoma of skin of left upper limb, including shoulder |
| C4491 | Basal cell carcinoma of skin, unspecified |
| C50411 | Malignant neoplasm of upper-outer quadrant of right female breast |
| C50412 | Malignant neoplasm of upper-outer quadrant of left female breast |
| C50811 | Malignant neoplasm of overlapping sites of right female breast |
| C50812 | Malignant neoplasm of overlapping sites of left female breast |
| C50911 | Malignant neoplasm of unspecified site of right female breast |
| C50912 | Malignant neoplasm of unspecified site of left female breast |
| C50919 | Malignant neoplasm of unspecified site of unspecified female breast |
| C541 | Malignant neoplasm of endometrium |
| C569 | Malignant neoplasm of unspecified ovary |
| C61 | Malignant neoplasm of prostate |
| C719 | Malignant neoplasm of brain, unspecified |
| C73 | Malignant neoplasm of thyroid gland |
| C773 | Secondary & unspec malignant neoplasm of axilla & upper limb lymph nodes |
| C787 | Secondary malignant neoplasm of liver and intrahepatic bile duct |
| C7931 | Secondary malignant neoplasm of brain |
| C7951 | Secondary malignant neoplasm of bone |
| C801 | Malignant (primary) neoplasm, unspecified |
| C8590 | Non-Hodgkin lymphoma, unspecified, unspecified site |
| D0439 | Carcinoma in situ of skin of other parts of face |
| D0511 | Intraductal carcinoma in situ of right breast |
| D0512 | Intraductal carcinoma in situ of left breast |
| D069 | Carcinoma in situ of cervix, unspecified |
| D1801 | Hemangioma of skin and subcutaneous tissue |
| D320 | Benign neoplasm of cerebral meninges |
| D329 | Benign neoplasm of meninges, unspecified |
| D485 | Neoplasm of uncertain behavior of skin |
| D496 | Neoplasm of unspecified behavior of brain |
| D649 | Anemia, unspecified |
| D849 | Immunodeficiency, unspecified |
| E038 | Other specified hypothyroidism |
| E039 | Hypothyroidism, unspecified |
| E041 | Nontoxic single thyroid nodule |
| E042 | Nontoxic multinodular goiter |
| E049 | Nontoxic goiter, unspecified |
| E0500 | Thyrotoxicosis with diffuse goiter without thyrotoxic crisis or storm |
| E0590 | Thyrotoxicosis, unspecified without thyrotoxic crisis or storm |
| E063 | Autoimmune thyroiditis |
| E079 | Disorder of thyroid, unspecified |
| E1065 | Type 1 diabetes mellitus with hyperglycemia |
| E109 | Type 1 diabetes mellitus without complications |
| E1110 | Type 2 diabetes mellitus with ketoacidosis without coma |
| E1121 | Type 2 diabetes mellitus with diabetic nephropathy |
| E1122 | Type 2 diabetes mellitus with diabetic chronic kidney disease |
| E1129 | Type 2 diabetes mellitus with other diabetic kidney complication |
| E11319 | Type 2 diabetes mellitus w unspec diabetic retinopathy w/o macular edema |
| E113293 | Type 2 diabetes mellitus w mild nonprolif DR w/o macular edema, bilat |
| E113393 | Type 2 diabetes mellitus w mod nonprolif DR w/o macular edema, bilateral |
| E113513 | Type 2 diabetes mellitus w prolif diab retinop w macular edema, bilat |
| E113593 | Type 2 diabetes mellitus with prolif DR w/o macular edema, bilateral |
| E1136 | Type 2 diabetes mellitus with diabetic cataract |
| E1139 | Type 2 diabetes mellitus with other diabetic ophthalmic complication |
| E1140 | Type 2 diabetes mellitus with diabetic neuropathy, unspecified |
| E1142 | Type 2 diabetes mellitus with diabetic polyneuropathy |
| E1151 | Type 2 diabetes mellitus w diabetic peripheral angiopathy w/o gangrene |
| E1159 | Type 2 diabetes mellitus with other circulatory complications |
| E11621 | Type 2 diabetes mellitus with foot ulcer |
| E11649 | Type 2 diabetes mellitus with hypoglycemia without coma |
| E1165 | Type 2 diabetes mellitus with hyperglycemia |
| E1169 | Type 2 diabetes mellitus with other specified complication |
| E118 | Type 2 diabetes mellitus with unspecified complications |
| E119 | Type 2 diabetes mellitus without complications |
| E139 | Other specified diabetes mellitus without complications |
| E230 | Hypopituitarism |
| E282 | Polycystic ovarian syndrome |
| E291 | Testicular hypofunction |
| E538 | Deficiency of other specified B group vitamins |
| E611 | Iron deficiency |
| E6601 | Morbid (severe) obesity due to excess calories |
| E6609 | Other obesity due to excess calories |
| E663 | Overweight |
| E668 | Other obesity |
| E669 | Obesity, unspecified |
| E7800 | Pure hypercholesterolemia, unspecified |
| E7801 | Familial hypercholesterolemia |
| E781 | Pure hyperglyceridemia |
| E782 | Mixed hyperlipidemia |
| E7849 | Other hyperlipidemia |
| E785 | Hyperlipidemia, unspecified |
| E786 | Lipoprotein deficiency |
| E8352 | Hypercalcemia |
| E860 | Dehydration |
| E871 | Hypo-osmolality and hyponatremia |
| E875 | Hyperkalemia |
| E876 | Hypokalemia |
| E8881 | Metabolic syndrome and other insulin resistance |
| E890 | Postprocedural hypothyroidism |
| F209 | Schizophrenia, unspecified |
| F250 | Schizoaffective disorder, bipolar type |
| F259 | Schizoaffective disorder, unspecified |
| F29 | Unspecified psychosis not due to substance or known physio condition |
| F312 | Bipolar disorder, current episode manic severe with psychotic features |
| F3131 | Bipolar disorder, current episode depressed, mild |
| F3132 | Bipolar disorder, current episode depressed, moderate |
| F314 | Bipolar disord, current episode depressed, severe, w/o psychotic feature |
| F3181 | Bipolar II disorder |
| F319 | Bipolar disorder, unspecified |
| F329 | Major depressive disorder, single episode, unspecified |
| F331 | Major depressive disorder, recurrent, moderate |
| F332 | Major depressive disorder, recurrent severe without psychotic features |
| F39 | Unspecified mood [affective] disorder |
| F640 | Transsexualism |
| F649 | Gender identity disorder, unspecified |
| F840 | Autistic disorder |
| G20 | Parkinsons disease |
| G245 | Blepharospasm |
| G250 | Essential tremor |
| G2581 | Restless legs syndrome |
| G3184 | Mild cognitive impairment of uncertain or unknown etiology |
| G35 | Multiple sclerosis |
| G40109 | Local-related Sx epilepsy & epileptic syndrome w SPS, not intract w/o SE |
| G40209 | Local-related Sx epilepsy & epileptic syndrome w CPS, not intract w/o SE |
| G40309 | Generalized idio epilepsy & epileptic syndrome, not intractable w/o SE |
| G40409 | Other generalized epilepsy & epileptic syndromes, not intractable w/o SE |
| G4089 | Other seizures |
| G40909 | Epilepsy, unspecified, not intractable, without status epilepticus |
| G459 | Transient cerebral ischemic attack, unspecified |
| G4700 | Insomnia, unspecified |
| G4709 | Other insomnia |
| G4710 | Hypersomnia, unspecified |
| G4730 | Sleep apnea, unspecified |
| G4733 | Obstructive sleep apnea (adult) (pediatric) |
| G479 | Sleep disorder, unspecified |
| G5600 | Carpal tunnel syndrome, unspecified upper limb |
| G5601 | Carpal tunnel syndrome, right upper limb |
| G5602 | Carpal tunnel syndrome, left upper limb |
| G5603 | Carpal tunnel syndrome, bilateral upper limbs |
| G629 | Polyneuropathy, unspecified |
| G8918 | Other acute postprocedural pain |
| G8929 | Other chronic pain |
| G893 | Neoplasm related pain (acute) (chronic) |
| G894 | Chronic pain syndrome |
| G930 | Cerebral cysts |
| G9340 | Encephalopathy, unspecified |
| G9389 | Other specified disorder of brain |
| G939 | Disorder of brain, unspecified |
| H04123 | Dry eye syndrome of bilateral lacrimal glands |
| H1045 | Other chronic allergic conjunctivitis |
| H35033 | Hypertensive retinopathy, bilateral |
| H353131 | Nonexudative age-related macular degen, bilateral, early dry stage |
| H35363 | Drusen (degenerative) of macula, bilateral |
| H35371 | Puckering of macula, right eye |
| H35372 | Puckering of macula, left eye |
| H35373 | Puckering of macula, bilateral |
| H35413 | Lattice degeneration of retina, bilateral |
| H40003 | Preglaucoma, unspecified, bilateral |
| H40013 | Open angle with borderline findings, low risk, bilateral |
| H40023 | Open angle with borderline findings, high risk, bilateral |
| H40033 | Anatomical narrow angle, bilateral |
| H40053 | Ocular hypertension, bilateral |
| H401130 | Primary open-angle glaucoma, bilateral, stage unspecified |
| H401131 | Primary open-angle glaucoma, bilateral, mild stage |
| H401132 | Primary open-angle glaucoma, bilateral, moderate stage |
| H409 | Unspecified glaucoma |
| H5213 | Myopia, bilateral |
| H524 | Presbyopia |
| H538 | Other visual disturbances |
| I10 | Essential (primary) hypertension |
| I110 | Hypertensive heart disease with heart failure |
| I119 | Hypertensive heart disease without heart failure |
| I120 | Hypertensive chronic kidney disease stage 5 or end stage renal disease |
| I129 | Hypertensive chronic kidney disease stage 1 through stage 4 or unspec |
| I130 | HTN heart & chr kidney disease w HF & stage 1 through 4 CKD or unspec |
| I160 | Hypertensive urgency |
| I161 | Hypertensive emergency |
| I200 | Unstable angina |
| I208 | Other forms of angina pectoris |
| I209 | Angina pectoris, unspecified |
| I213 | ST elevation (STEMI) myocardial infarction of unspecified site |
| I214 | Non-ST elevation (NSTEMI) myocardial infarction |
| I249 | Acute ischemic heart disease, unspecified |
| I2510 | Atherosclerotic heart disease native coronary artery w/o angina pectoris |
| I25110 | ASHD of native coronary artery with unstable angina pectoris |
| I25118 | ASHD of native coronary artery with other forms of angina pectoris |
| I25119 | ASHD of native coronary artery with unspecified angina pectoris |
| I252 | Old myocardial infarction |
| I255 | Ischemic cardiomyopathy |
| I340 | Nonrheumatic mitral (valve) insufficiency |
| I341 | Nonrheumatic mitral (valve) prolapse |
| I350 | Nonrheumatic aortic (valve) stenosis |
| I351 | Nonrheumatic aortic (valve) insufficiency |
| I420 | Dilated cardiomyopathy |
| I428 | Other cardiomyopathies |
| I429 | Cardiomyopathy, unspecified |
| I480 | Paroxysmal atrial fibrillation |
| I4891 | Unspecified atrial fibrillation |
| I5020 | Unspecified systolic (congestive) heart failure |
| I5022 | Chronic systolic (congestive) heart failure |
| I509 | Heart failure, unspecified |
| I517 | Cardiomegaly |
| I6389 | Other cerebral infarction |
| I639 | Cerebral infarction, unspecified |
| I6523 | Occlusion and stenosis of bilateral carotid arteries |
| I671 | Cerebral aneurysm, nonruptured |
| I6782 | Cerebral ischemia |
| I700 | Atherosclerosis of aorta |
| I7300 | Raynauds syndrome without gangrene |
| I739 | Peripheral vascular disease, unspecified |
| I959 | Hypotension, unspecified |
| J189 | Pneumonia, unspec organism |
| J42 | Unspecified chronic bronchitis |
| J439 | Emphysema, unspecified |
| J441 | Chronic obstructive pulmonary disease with (acute) exacerbation |
| J449 | Chronic obstructive pulmonary disease, unspecified |
| J4520 | Mild intermittent asthma, uncomplicated |
| J4521 | Mild intermittent asthma with (acute) exacerbation |
| J4530 | Mild persistent asthma, uncomplicated |
| J4531 | Mild persistent asthma with (acute) exacerbation |
| J4540 | Moderate persistent asthma, uncomplicated |
| J4541 | Moderate persistent asthma with (acute) exacerbation |
| J4550 | Severe persistent asthma, uncomplicated |
| J45901 | Unspecified asthma with (acute) exacerbation |
| J45909 | Unspecified asthma, uncomplicated |
| J45990 | Exercise induced bronchospasm |
| J45991 | Cough variant asthma |
| J45998 | Other asthma |
| J811 | Chronic pulmonary edema |
| J90 | Pleural effusion, not elsewhere classified |
| J9600 | Acute respiratory failure, unspec whether hypoxia or hypercapnia |
| J9601 | Acute respiratory failure with hypoxia |
| J9690 | Respiratory failure, unspecified, unspec whether hypoxia or hypercapnia |
| J9811 | Atelectasis |
| J984 | Other disorders of lung |
| K219 | Gastro-esophageal reflux disease without esophagitis |
| K2950 | Unspecified chronic gastritis without bleeding |
| K5000 | Crohns disease of small intestine without complications |
| K5010 | Crohns disease of large intestine without complications |
| K5080 | Crohns disease of both small and large intestine without complications |
| K5090 | Crohns disease, unspecified, without complications |
| K5100 | Ulcerative (chronic) pancolitis without complications |
| K5120 | Ulcerative (chronic) proctitis without complications |
| K5150 | Left sided colitis without complications |
| K5190 | Ulcerative colitis, unspecified, without complications |
| K529 | Noninfective gastroenteritis and colitis, unspecified |
| K5900 | Constipation, unspecified |
| K6389 | Other specified disease of intestine |
| K7460 | Unspecified cirrhosis of liver |
| K760 | Fatty (change of) liver, not elsewhere classified |
| K7689 | Other specified disease of liver |
| K769 | Liver disease, unspecified |
| K922 | Gastrointestinal hemorrhage, unspecified |
| L309 | Dermatitis, unspecified |
| L400 | Psoriasis vulgaris |
| L4050 | Arthropathic psoriasis, unspecified |
| L408 | Other psoriasis |
| L409 | Psoriasis, unspecified |
| L570 | Actinic keratosis |
| L820 | Inflamed seborrheic keratosis |
| L851 | Acquired keratosis [keratoderma] palmaris et plantaris |
| L858 | Other specified epidermal thickening |
| M0579 | Rheum arthritis w rheum factor of multiple site w/o organ or syst involv |
| M059 | Rheumatoid arthritis with rheumatoid factor, unspecified |
| M0609 | Rheumatoid arthritis without rheumatoid factor, multiple sites |
| M064 | Inflammatory polyarthropathy |
| M069 | Rheumatoid arthritis, unspecified |
| M129 | Arthropathy, unspecified |
| M130 | Polyarthritis, unspecified |
| M150 | Primary generalized (osteo)arthritis |
| M159 | Polyosteoarthritis, unspecified |
| M160 | Bilateral primary osteoarthritis of hip |
| M1611 | Unilateral primary osteoarthritis, right hip |
| M1612 | Unilateral primary osteoarthritis, left hip |
| M170 | Bilateral primary osteoarthritis of knee |
| M1710 | Unilateral primary osteoarthritis, unspecified knee |
| M1711 | Unilateral primary osteoarthritis, right knee |
| M1712 | Unilateral primary osteoarthritis, left knee |
| M179 | Osteoarthritis of knee, unspecified |
| M180 | Bilateral primary osteoarthritis of first carpometacarpal joints |
| M1811 | Unilat primary osteoarthritis of first carpometacarpal joint, right hand |
| M1812 | Unilat primary osteoarthritis of first carpometacarpal joint, left hand |
| M19011 | Primary osteoarthritis, right shoulder |
| M19012 | Primary osteoarthritis, left shoulder |
| M19031 | Primary osteoarthritis, right wrist |
| M19041 | Primary osteoarthritis, right hand |
| M19042 | Primary osteoarthritis, left hand |
| M19049 | Primary osteoarthritis, unspecified hand |
| M19071 | Primary osteoarthritis, right ankle and foot |
| M19072 | Primary osteoarthritis, left ankle and foot |
| M1990 | Unspecified osteoarthritis, unspecified site |
| M25461 | Effusion, right knee |
| M25462 | Effusion, left knee |
| M2550 | Pain in unspecified joint |
| M25511 | Pain in right shoulder |
| M25512 | Pain in left shoulder |
| M25531 | Pain in right wrist |
| M25532 | Pain in left wrist |
| M25551 | Pain in right hip |
| M25552 | Pain in left hip |
| M25561 | Pain in right knee |
| M25562 | Pain in left knee |
| M25569 | Pain in unspecified knee |
| M25571 | Pain in right ankle & joints right foot |
| M25572 | Pain in left ankle & joints left foot |
| M26601 | Right temporomandibular joint disorder, unspecified |
| M26602 | Left temporomandibular joint disorder, unspecified |
| M26603 | Bilateral temporomandibular joint disorder, unspecified |
| M26609 | Unspecified temporomandibular joint disorder, unspecified side |
| M26621 | Arthralgia of right temporomandibular joint |
| M26622 | Arthralgia of left temporomandibular joint |
| M26623 | Arthralgia of bilateral temporomandibular joint |
| M26629 | Arthralgia of temporomandibular joint, unspecified side |
| M329 | Systemic lupus erythematosus, unspecified |
| M3500 | Sjogren syndrome, unspecified |
| M3501 | Sjogren syndrome with keratoconjunctivitis |
| M419 | Scoliosis, unspecified |
| M459 | Ankylosing spondylitis of unspecified sites in spine |
| M461 | Sacroiliitis, not elsewhere classified |
| M4712 | Other spondylosis with myelopathy, cervical region |
| M4722 | Other spondylosis with radiculopathy, cervical region |
| M4726 | Other spondylosis with radiculopathy, lumbar region |
| M47812 | Spondylosis without myelopathy or radiculopathy, cervical region |
| M47814 | Spondylosis without myelopathy or radiculopathy, thoracic region |
| M47816 | Spondylosis without myelopathy or radiculopathy, lumbar region |
| M47817 | Spondylosis without myelopathy or radiculopathy, lumbosacral region |
| M47892 | Other spondylosis, cervical region |
| M47896 | Other spondylosis, lumbar region |
| M4802 | Spinal stenosis, cervical region |
| M48061 | Spinal stenosis, lumbar region without neurogenic claudication |
| M533 | Sacrococcygeal disorders, not elsewhere classified |
| M5412 | Radiculopathy, cervical region |
| M5416 | Radiculopathy, lumbar region |
| M5417 | Radiculopathy, lumbosacral region |
| M542 | Cervicalgia |
| M545 | Low back pain |
| M5450 | Low back pain, unspecified |
| M549 | Dorsalgia, unspecified |
| M722 | Plantar fascial fibromatosis |
| M7918 | Myalgia, other site |
| M79604 | Pain in right leg |
| M79605 | Pain in left leg |
| M79641 | Pain in right hand |
| M79642 | Pain in left hand |
| M79644 | Pain in right finger(s) |
| M79671 | Pain in right foot |
| M79672 | Pain in left foot |
| M7989 | Other specified soft tissue disorder |
| N1330 | Unspecified hydronephrosis |
| N179 | Acute kidney failure, unspecified |
| N182 | Chronic kidney disease, stage 2 (mild) |
| N183 | Chronic kidney disease, stage 3 (moderate) |
| N1830 | Chronic kidney disease, stage 3 unspecified |
| N1831 | Chronic kidney disease, stage 3a |
| N1832 | Chronic kidney disease, stage 3b |
| N184 | Chronic kidney disease, stage 4 (severe) |
| N185 | Chronic kidney disease, stage 5 |
| N186 | End stage renal disease |
| N189 | Chronic kidney disease, unspecified |
| N281 | Cyst of kidney, acquired |
| N2889 | Other specified disorders of kidney & ureter |
| N289 | Disorder of kidney and ureter, unspecified |
| N390 | Urinary tract infection, site not specified |
| N400 | Benign prostatic hyperplasia without lower urinary tract symptoms |
| N401 | Benign prostatic hyperplasia with lower urinary tract symptoms |
| N6310 | Unspecified lump in the right breast, unspecified quadrant |
| N6320 | Unspecified lump in the left breast, unspecified quadrant |
| N6489 | Other specified disorder of breast |
| N870 | Mild cervical dysplasia |
| N871 | Moderate cervical dysplasia |
| N879 | Dysplasia of cervix uteri, unspecified |
| N898 | Other specified noninflammatory disorders of vagina |
| N950 | Postmenopausal bleeding |
| P8381 | Umbilical granuloma |
| P925 | Neonatal difficulty in feeding at breast |
| Q210 | Ventricular septal defect |
| Q211 | Atrial septal defect |
| Q231 | Congenital insufficiency of aortic valve |
| Q825 | Congenital non-neoplastic nevus |
| Q828 | Other specified congenital malformations of skin |
| R000 | Tachycardia, unspecified |
| R001 | Bradycardia, unspecified |
| R002 | Palpitations |
| R011 | Cardiac murmur, unspecified |
| R030 | Elevated blood-pressure reading, without diagnosis of hypertension |
| R0600 | Dyspnea, unspecified |
| R0602 | Shortness of breath |
| R0609 | Other forms of dyspnea |
| R062 | Wheezing |
| R0683 | Snoring |
| R072 | Precordial pain |
| R0781 | Pleurodynia |
| R0789 | Oth chest pain |
| R079 | Chest pain, unspecified |
| R0902 | Hypoxemia |
| R0989 | Other specified symptoms and signs involv circulatory and resp systems |
| R1010 | Upper abdominal pain, unspecified |
| R1011 | Right upper quadrant pain |
| R1012 | Left upper quadrant pain |
| R1013 | Epigastric pain |
| R102 | Pelvic and perineal pain |
| R1030 | Lower abdominal pain, unspecified |
| R1031 | Right lower quadrant pain |
| R1032 | Left lower quadrant pain |
| R1084 | Generalized abdominal pain |
| R109 | Unspecified abdominal pain |
| R110 | Nausea |
| R1110 | Vomiting, unspecified |
| R112 | Nausea with vomiting, unspecified |
| R12 | Heartburn |
| R1310 | Dysphagia, unspecified |
| R140 | Abdominal distension (gaseous) |
| R160 | Hepatomegaly, not elsewhere classified |
| R17 | Unspecified jaundice |
| R188 | Other ascites |
| R1900 | Intra-abdominal and pelvic swelling, mass and lump, unspecified site |
| R194 | Change in bowel habit |
| R195 | Other fecal abnormalities |
| R197 | Diarrhea, unspecified |
| R198 | Other specified symptoms and signs involv digestive system and abdomen |
| R200 | Anesthesia of skin |
| R202 | Paresthesia of skin |
| R208 | Other disturbances of skin sensation |
| R21 | Rash and other nonspecific skin eruption |
| R220 | Localized swelling, mass and lump, head |
| R221 | Localized swelling, mass and lump, neck |
| R222 | Localized swelling, mass and lump, trunk |
| R2231 | Localized swelling, mass and lump, right upper limb |
| R2232 | Localized swelling, mass and lump, left upper limb |
| R2241 | Localized swelling, mass and lump, right lower limb |
| R2242 | Localized swelling, mass and lump, left lower limb |
| R229 | Localized swelling, mass and lump, unspecified |
| R232 | Flushing |
| R238 | Other skin change |
| R251 | Tremor, unspecified |
| R29818 | Other symptoms & signs involving the nervous system |
| R29898 | Other symptoms & signs involving the musculoskeletal system |
| R310 | Gross hematuria |
| R3129 | Other microscopic hematuria |
| R319 | Hematuria, unspecified |
| R410 | Disorientation, unspecified |
| R413 | Other amnesia |
| R4182 | Altered mental status, unspecified |
| R42 | Dizziness and giddiness |
| R45851 | Suicidal ideations |
| R509 | Fever, unspecified |
| R519 | Headache, unspecified |
| R52 | Pain, unspecified |
| R531 | Weakness |
| R5381 | Other malaise |
| R5382 | Chronic fatigue, unspecified |
| R5383 | Other fatigue |
| R55 | Syncope and collapse |
| R569 | Unspecified convulsions |
| R590 | Localized enlarged lymph nodes |
| R591 | Generalized enlarged lymph nodes |
| R600 | Localized edema |
| R609 | Edema, unspecified |
| R61 | Generalized hyperhidrosis |
| R630 | Anorexia |
| R633 | Feeding difficulties |
| R6330 | Feeding difficulties, unspecified |
| R6339 | Other feeding difficulties |
| R634 | Abnormal weight loss |
| R635 | Abnormal weight gain |
| R636 | Underweight |
| R6882 | Decreased libido |
| R6889 | Other general symptoms & signs |
| R69 | Illness, unspecified |
| R7301 | Impaired fasting glucose |
| R7302 | Impaired glucose tolerance (oral) |
| R7303 | Prediabetes |
| R7309 | Other abnormal glucose |
| R739 | Hyperglycemia, unspecified |
| R7401 | Elevation of levels of liver transaminase levels |
| R748 | Abnormal levels of other serum enzymes |
| R760 | Raised antibody titer |
| R768 | Other specified abnormal immunological findings in serum |
| R7881 | Bacteremia |
| R7989 | Other specified abnormal finding of blood chemistry |
| R809 | Proteinuria, unspecified |
| R8290 | Unspecified abnormal findings in urine |
| R87612 | Low grade squamous intraepith lesion cytologic smear of cervix (LGSIL) |
| R87613 | High grade squamous intraepith lesion cytologic smear of cervix (HGSIL) |
| R87619 | Unspecified abnormal cytological findings in specimens from cervix uteri |
| R9089 | Other abnormal finding on diagnostic imaging of central nervous system |
| R911 | Solitary pulmonary nodule |
| R918 | Other nonspecific abnormal finding of lung field |
| R921 | Mammographic calcification found on diagnostic imaging of breast |
| R922 | Inconclusive mammogram |
| R928 | Other abnormal and inconclusive findings on diagnostic imaging of breast |
| R930 | Abnormal findings on diagnostic imaging of skull and head, NEC |
| R935 | Abnormal findings on diagnostic imaging oth abd regions, incl retroperit |
| R9389 | Abnormal findings on diagnostic imag of other specified body structures |
| R9431 | Abnormal electrocardiogram [ECG] [EKG] |
| R945 | Abnormal results of liver function studies |
| R9720 | Elevated prostate specific antigen [PSA] |
| T148XXA | Other injury of unspecified body region, initial encounter |
| T7840XA | Allergy, unspecified, initial encounter |
| U071 | COVID-19 |
| Z0101 | Encounter for examination of eyes and vision with abnormal findings |
| Z0110 | Encounter for examination of ears and hearing without abnormal findings |
| Z01810 | Encounter for preprocedural cardiovascular examination |
| Z01811 | Encounter for preprocedural respiratory examination |
| Z01812 | Encounter for preprocedural laboratory examination |
| Z01818 | Encounter for other preprocedural examination |
| Z0189 | Encounter for other specified special examination |
| Z029 | Encounter for administrative examinations, unspecified |
| Z03818 | Encounter observation suspect expos to biological agents NEC ruled out |
| Z0389 | Encounter for observation for oth suspect disease & conditions ruled out |
| Z08 | Encounter follow-up exam after completed Tx for other than mal neoplasm |
| Z09 | Encounter for follow-up after completed Tx for condition not mal neopl |
| Z1211 | Encounter for screening for malignant neoplasm of colon |
| Z1341 | Encounter for autism screening |
| Z135 | Encounter for screening for eye and ear disorders |
| Z206 | Contact w & (suspected) exposure to human immunodeficiency virus [HIV] |
| Z20822 | Contact with and (suspected) exposure to COVID-19 |
| Z20828 | Contact with and (suspected) exposure to oth viral communicable diseases |
| Z21 | Asymptomatic human immunodeficiency virus [HIV] infection status |
| Z421 | Encounter for breast reconstruction following mastectomy |
| Z452 | Encounter for adjustment and management of vascular access device |
| Z4659 | Encounter for fitting & adjust of other gastrointest appliance & device |
| Z4682 | Encounter for fitting and adjustment of non-vascular catheter |
| Z471 | Aftercare following joint replacement surgery |
| Z4789 | Encounter for oth orthopedic aftercare |
| Z4802 | Encounter for removal of sutures |
| Z483 | Aftercare following surgery for neoplasm |
| Z48810 | Encounter for surgical aftercare following surgery on the sense organs |
| Z48812 | Encounter for surgical aftercare following surgery on circulatory system |
| Z48815 | Encounter for surgical aftercare following surgery on digestive system |
| Z48816 | Encounter for surgical aftercare follow surgery on genitourinary system |
| Z48817 | Encounter for surgical aftercare follow surgery on skin & subsq tissue |
| Z4889 | Encounter for other specified surgical aftercare |
| Z510 | Encounter for antineoplastic radiation therapy |
| Z5111 | Encounter for antineoplastic chemotherapy |
| Z5112 | Encounter for antineoplastic immunotherapy |
| Z515 | Encounter for palliative care |
| Z5181 | Encounter for therapeutic drug level monitoring |
| Z5189 | Encounter for oth specified aftercare |
| Z6825 | Body mass index [BMI] 25.0-25.9, adult |
| Z6826 | Body mass index [BMI] 26.0-26.9, adult |
| Z6827 | Body mass index [BMI] 27.0-27.9, adult |
| Z6828 | Body mass index [BMI] 28.0-28.9, adult |
| Z6829 | Body mass index [BMI] 29.0-29.9, adult |
| Z6830 | Body mass index [BMI] 30.0-30.9, adult |
| Z6831 | Body mass index [BMI] 31.0-31.9, adult |
| Z6832 | Body mass index [BMI] 32.0-32.9, adult |
| Z6833 | Body mass index [BMI] 33.0-33.9, adult |
| Z6834 | Body mass index [BMI] 34.0-34.9, adult |
| Z6835 | Body mass index [BMI] 35.0-35.9, adult |
| Z6836 | Body mass index [BMI] 36.0-36.9, adult |
| Z6837 | Body mass index [BMI] 37.0-37.9, adult |
| Z6838 | Body mass index [BMI] 38.0-38.9, adult |
| Z6839 | Body mass index [BMI] 39.0-39.9, adult |
| Z6841 | Body mass index [BMI] 40.0-44.9, adult |
| Z6842 | Body mass index [BMI] 45.0-49.9, adult |
| Z6843 | Body mass index [BMI] 50.0-59.9, adult |
| Z6852 | Body mass index [BMI] pediatric, 5th percent to < 85th percent for age |
| Z6853 | Body mass index [BMI] pediatric, 85th percent to < 95th percent for age |
| Z6854 | Body mass index pediatric, 95th% for age to <120% of 95th% for age |
| Z711 | Person with feared health complaint in whom no diagnosis is made |
| Z7689 | Persons encountering health services in other specified circumstance |
| Z85038 | Personal history of other malignant neoplasm of large intestine |
| Z853 | Personal history of malignant neoplasm of breast |
| Z8546 | Personal history of malignant neoplasm of prostate |
| Z85820 | Personal history of malignant melanoma of skin |
| Z85828 | Personal history of other malignant neoplasm of skin |
| Z85850 | Personal history of malignant neoplasm of thyroid |
| Z951 | Presence of aortocoronary bypass graft |
| Z955 | Presence of coronary angioplasty implant and graft |
| Z95810 | Presence of automatic (implantable) cardiac defibrillator |
| Z961 | Presence of intraocular lens |
| Z96641 | Presence of right artificial hip joint |
| Z96642 | Presence of left artificial hip joint |
| Z96651 | Presence of right artificial knee joint |
| Z96652 | Presence of left artificial knee joint |
| Z98890 | Other specified postprocedural states |
